# An Internet of Things–Based Audio and Radio Connected System Supporting Older Adults With Physical and Cognitive Health Challenges: Qualitative Stakeholder-Informed Design Study

**DOI:** 10.2196/76341

**Published:** 2026-04-29

**Authors:** Jia Liu, Xiaomeng Wang, Bianca Shieu, Deanna Dolores Garcia, Amy Vondenberger, Jingye Xu, Yuntong Zhang, Mahathir Monjur, Wei Wang, Shahriar Nirjon, Robert Svatek, Mitzi Gonzales, Neela Patel, Lixin Song

**Affiliations:** 1 School of Nursing The University of Texas Health Science Center at San Antonio San Antonio, TX United States; 2 College of AI, Cyber and Computing The University of Texas at San Antonio San Antonio, TX United States; 3 Computer Science Department Prairie View A&M University Prairie View, TX United States; 4 Department of Computer Science University of North Carolina at Chapel Hill Chapel Hill, NC United States; 5 Department of Urology The University of Texas Health Science Center at San Antonio San Antonio, TX United States; 6 Department of Neurology Cedars-Sinai Medical Center Los Angeles, CA United States; 7 Department of Family and Community Medicine The University of Texas Health Science Center at San Antonio San Antonio, TX United States

**Keywords:** Alzheimer disease, assistive technology, cancer, Internet of Things, older adult, self-management, stakeholder engagement

## Abstract

**Background:**

Older adults managing chronic illnesses, such as cancer and Alzheimer disease and related dementias (ADRD), often experience significant physical or cognitive impairments that hinder daily activities and increase caregiver burden. Smart Internet of Things (IoT) technologies offer promising solutions by enabling passive monitoring, timely reminders, and personalized support at home. However, these technologies must be carefully tailored to accommodate users’ individualized needs and preferences.

**Objective:**

This formative qualitative study aimed to explore stakeholder perspectives, including patients, caregivers, health care providers, and technical experts, on the use of smart home–based IoT systems to support chronic illness management. The goal was to inform the early development of the audio and radio connected (AURA) system, an IoT prototype integrating Wi-Fi sensing, wearable trackers, and voice-assistive features.

**Methods:**

Semistructured interviews were conducted with 6 patients who underwent postostomy creation for colorectal or bladder cancer treatment and 5 patients with ADRD and their caregivers. Input from additional stakeholders, including 2 health care providers, 2 community health workers, and 2 computer scientists, was also included in the report. Stakeholders reviewed a demonstration video depicting the conceptual features of the AURA system. Interviews explored stakeholders’ needs and preferences for using such systems. Thematic analysis was guided by the extended Unified Theory of Acceptance and Use of Technology 2 (UTAUT2) framework, with 5 adapted constructs: performance expectancy, effort expectancy, social influence, facilitating conditions, and hedonic motivation and habit.

**Results:**

Stakeholders identified distinct yet complementary needs across populations. Patients with cancer emphasized physical health monitoring, integration with health care systems, and customization; ADRD stakeholders prioritized routine support, emotional engagement, and simplicity; caregivers and clinicians emerged as key influencers of adoption. Barriers included privacy concerns, technology literacy, and fatigue, while facilitators included perceived caregiving support, streamlined interfaces, and electronic health record integration. Patients with cancer focused on motivational cues for physical activity, while emotional engagement and habit were more prominent for ADRD users.

**Conclusions:**

Stakeholder insights underscore the importance of designing adaptable, user-centered IoT systems that reflect the varied capabilities and care needs of older adults with chronic illnesses. These findings informed the design of the AURA prototype and highlighted theoretical considerations for technology acceptance in health care. Future work will test AURA in real-world settings to evaluate usability, acceptability, and clinical relevance.

## Introduction

### Physical and Cognitive Health Challenges in Older Adults With Chronic Illnesses

Physical and cognitive health challenges associated with chronic illnesses can significantly impact the daily lives of older adults and their caregivers. The challenges can impair patients’ ability to maintain independence, manage health effectively, and ensure safety at home [1]. Physical impairments, such as limited mobility or fatigue, can hinder basic functions, including walking, eating, and using medical equipment [2]. Cognitive impairments, including memory loss and difficulties in decision-making, disrupt essential routines and increase the reliance on caregivers for activities of daily living (ADLs) [3]. The limitations contribute to heightened caregiver burden and reduce patients’ ability to manage their health at home safely.

### Physical and Cognitive Challenges in Patients With Cancer and Alzheimer Disease and Related Dementias

Among older adults with chronic illnesses, 2 representative groups particularly affected by the aforementioned challenges are patients with cancer recovering from ostomy surgeries and patients living with Alzheimer disease and related dementias (ADRD). Patients undergoing bladder or colorectal cancer treatment with ostomy placement often experience physical limitations that impede recovery and elevate the risks of dehydration [4], infection [5], or complications related to stoma care and ostomy bag management [6]. In contrast, patients with ADRD primarily struggle with cognitive impairments that interfere with daily routines such as eating, medication adherence, and hygiene, which often require constant supervision and prompting [7]. In both populations, unmet care needs in the home environment can threaten quality of life (QoL) and increase strain on caregivers.

### Applications of Internet of Things in Supporting Physical and Cognitive Challenges

As more older adults continue to age in place despite declines in physical and cognitive health, there is a growing need for home-based solutions that promote safety, autonomy, and the continuity of care [8]. Smart Internet of Things (IoT) technologies offer a promising pathway to meet these needs by embedding real-time monitoring and assistive functions into the living environment. These technologies comprise networks of connected devices and sensors that communicate and share data to enable automated, real-time responses tailored to individual needs [9].

IoT technologies have been applied to support various health needs for older adults. For those with physical impairment, IoT-enabled monitoring systems can track movement [10], detect falls [9], and manage medical devices such as ostomy bags or health sensors [11,12]. For individuals with cognitive impairment, IoT-connected voice assistants can deliver personalized reminders for tasks such as taking medications, eating meals, or attending appointments [13]. Meanwhile, health monitoring systems empowered by IoT, such as Wi-Fi sensing or motion detectors, can noninvasively detect deviations in daily routines, potential safety issues, or the need for caregiver support [14]. Additionally, IoT systems can foster social interaction and reduce isolation and loneliness by providing reminders for social activities or virtual interactions through web-based chat, videoconferencing, group chat, and email [15]. These functionalities can enhance daily functioning, reduce risks, and promote emotional well-being in the home setting.

### Designing IoT for Individualized Care Needs

Despite the potential of smart IoT technologies to support patients and their caregivers, designing individualized systems to address complex care needs remains difficult [16]. Many existing technologies are not adequately tailored to the diverse functional and cognitive profiles of older adults. Solutions must balance complexity and simplicity [17] while maintaining privacy [18], supporting caregiver roles [19], and ensuring ease of use [20]. For example, patients with cancer may benefit from activity tracking and hydration prompts, while patients with ADRD may need intuitive, voice-activated routines that are accessible with their impaired cognitive ability.

To support the development of a smart IoT-based system prototype that addresses complex needs, this study aims to explore stakeholder needs and preferences for using smart home monitoring and assistive technologies. By gathering input from stakeholders, including patients, caregivers, health care providers, and technologists, we provide early formative evidence to inform the design of inclusive and user-centered systems. Specifically, this study contributes to the literature by (1) applying an extended and adapted framework to examine the perceived value, usability, and adoption barriers of smart IoT technologies among older adults with physical and cognitive impairments, (2) highlighting cross-population differences that should inform design tailoring, and (3) offering actionable recommendations to bridge the gap between user needs and technological design. These insights lay the groundwork for future development, refinement, and deployment of personalized IoT solutions that support aging in place and reduce caregiver burden.

## Methods

### Study Design

This study used a qualitative, exploratory design to collect stakeholder input, that is, the needs and preferences that would inform the early development of a smart home–based IoT system to support physical and cognitive health challenges. Data were collected through semistructured interviews with older adult patients and caregivers from 2 distinct populations representing physical and cognitive impairments, namely (1) patients with cancer who had ostomies and (2) patients with ADRD and their caregivers.

Involving both patients and caregivers in the early design process was critical to capturing a comprehensive understanding of home care needs, preferences for system use, and usability considerations [21]. Patients provide insight into their lived experiences and functional limitations, while caregivers contribute perspectives on feasibility, usability, and continuity of care, particularly relevant for people with ADRD, who may have limited decision-making capacity.

In addition to patients and caregivers, health care providers, community health workers, and computer scientists were also interviewed to offer broader perspectives on the clinical, community, and technical implications of developing a supportive IoT-based system.

### Stakeholders and Recruitment

Stakeholders enrolled in this study included the following:

1. Patients with cancer who had ostomies: eligible patients had a history of bladder or colorectal cancer treated with curative intent ostomy, were 18 years of age or older, and were able to read and speak English.
2. Patients with ADRD and their caregivers: eligible patients were diagnosed with mild cognitive impairment or early-stage ADRD, lived with a caregiver present at least 4 hours/day for at least 6 months, were 18 years of age or older, and had sufficient vision and speech to use a voice-assistive system.
3. Health care professionals and technical experts: a neuropsychologist, a geriatrician, 2 community health workers who provide direct care and service to patients with ADRD, and 2 computer scientists were recruited to offer broader perspectives on care needs and system development.

Patients with cancer were referred from the Oncology Clinic at the Mays Cancer Center, while patients with ADRD were referred by clinicians from the Geriatrics and Palliative Care Clinic, all of which are affiliated with the University of Texas Health Science Center at San Antonio. After confirming eligibility through a medical record review, the research team contacted potential patients and caregivers to schedule interviews.

### Setting and Procedures

**Figure 1:**
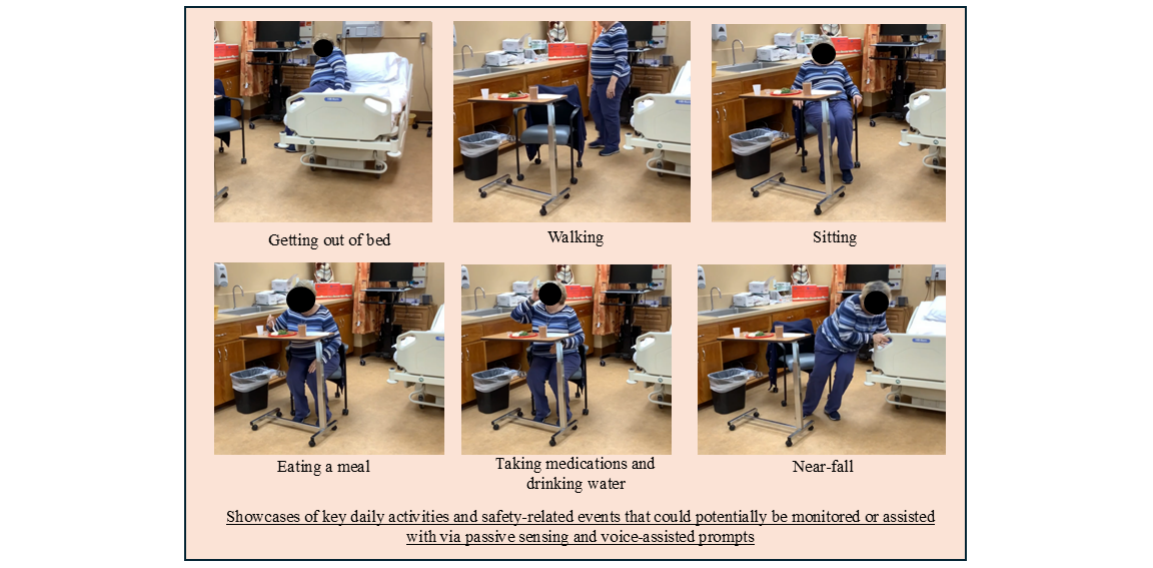
Simulated scenarios in the demonstration video.

Eligible patients and their caregivers participated in in-person interviews at the School of Nursing Community Simulation Lab. Interviews with nonpatient stakeholders, including health care providers, community health workers, and computer scientists, were conducted remotely via Zoom (Zoom Communications, Inc). Semistructured interviews were conducted by trained research assistants, lasting 30-45 minutes each. A researcher-developed interview guide was used to explore patients’ and caregivers’ needs and preferences for smart IoT technologies. To facilitate discussion, stakeholders were shown a brief demonstration video presenting a conceptual example of how a smart IoT-based system might function in the home environment. The video illustrated how simulated scenarios of daily activities and care challenges, such as monitoring mobility and prompting medication adherence, could be supported by passive activity detection and monitoring, as well as voice-assisted reminders (Figure 1). The purpose was to help stakeholders visualize potential applications in everyday care.

Interview questions were open-ended and neutrally phrased to reduce confirmation and social desirability bias [22]. The interviewer emphasized that the system was still under development and encouraged stakeholders to reflect on its relevance, including potential benefits and limitations, to their daily routines and caregiving needs. Interviewers avoided leading prompts to ensure that responses reflected authentic perspectives rather than agreement with the demonstrated concept. Sample interview questions included the following: “What are the benefits of using such a system?” and “What are the issues in relation to the use of such a system?” Moreover, questions such as “How do you feel this will replace what you are using now?” were included to prompt stakeholders to consider the potential role in their real-life setting, beyond what was shown in the video. All interviews were audio-recorded and transcribed verbatim for analysis.

### Initial Smart IoT System Concept: Audio and Radio Connected

**Figure 2:**
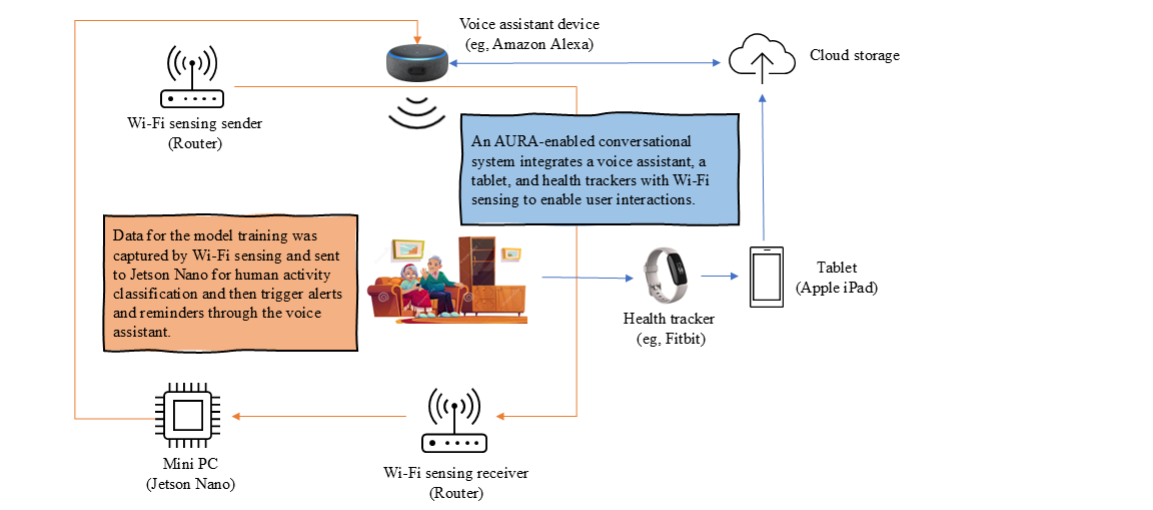
Initial audio and radio connected system concept.

To address the complex challenges associated with managing chronic illnesses at home, the research team conceptualized the audio and radio connected (AURA) system, an integrated smart IoT solution designed to support older adults with physical and cognitive impairments. Inspired by the needs of stakeholders identified during our previous Patient Reported Outcomes-Informed Symptom Management System (PRISMS) study [23], AURA aimed to provide individualized, real-time support, with a minimally intrusive design (Figure 2).

The envisioned system integrated 3 core components: Wi-Fi sensing, wearable health trackers, and voice-assistive technologies. At the heart of the system was a deep neural network [24] trained on Channel State Information (CSI) data collected from Wi-Fi sensing [25]. Using this radio frequency sensing approach, standard Wi-Fi routers and a deep learning model running on a Jetson Nano edge device could unobtrusively record and classify user activities, such as walking, sitting, or lying down, without requiring wearable devices [26]. This passive monitoring framework was particularly well-suited to individuals with limited mobility or cognitive impairments who may not consistently engage with wearable technology [27].

While the Wi-Fi sensing system supported user comfort and privacy, we enhanced this approach by incorporating wearable health trackers (eg, Fitbit, smart scales, and smart bottles). These devices collected continuous data on sleep quality, hydration, physical activity, and weight, which were critical for maintaining health during treatment recovery or cognitive decline. For example, patients with cancer who had ostomies were at increased risk for dehydration or delayed recovery due to inactivity. AURA could detect prolonged sedentary periods and issue reminders to drink water or engage in light movement. Similarly, patients with ADRD, who frequently forgot routine tasks, could benefit from scheduled voice prompts for meals, hydration, and medications.

Voice-assistive technologies, such as Alexa (Amazon), were envisioned as a central interface for patient engagement. These tools would deliver context-aware alerts and initiate personalized daily check-ins to assess users’ well-being. For example, voice prompts might ask whether a patient had eaten, how they were feeling, or if they were experiencing any discomfort (eg, changes in urine color or surgical site pain). By combining passive Wi-Fi sensing, health tracking, and engaged interaction, AURA supported both physical safety and cognitive functioning without requiring complex manual operation, making it especially valuable for older adults aging in place.

Overall, the AURA system concept was designed to enhance independence, promote safety, and reduce caregiver burden through an adaptable, user-centered approach to smart home care.

### Data Analysis

**Figure 3:**
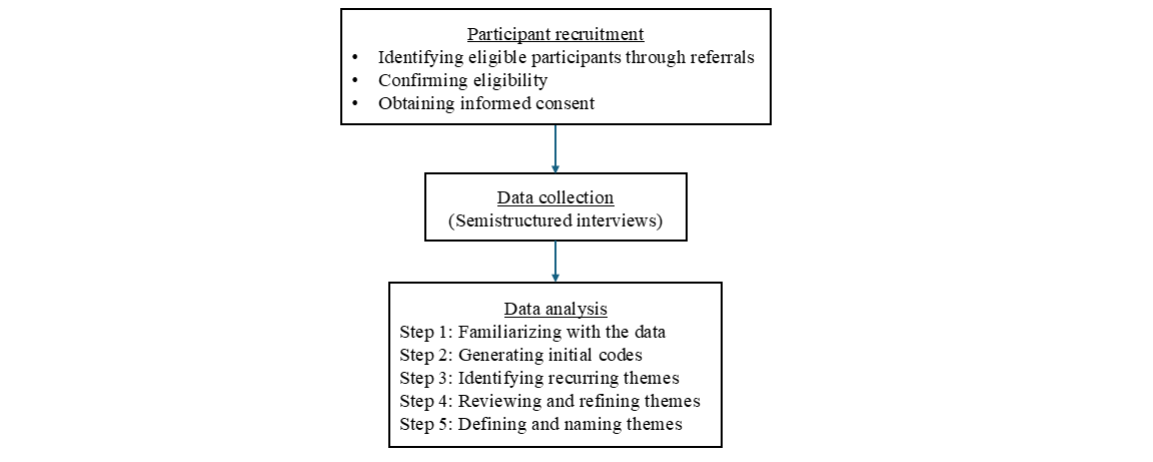
Study procedure.

We followed Braun and Clarke's thematic analysis framework [28] for data analysis, with additional guidance from the extended Unified Theory of Acceptance and Use of Technology 2 (UTAUT2) framework [29] (Figure 3). NVivo 14 (Lumivero) [30] was used to assist in organizing and coding the data. The thematic data analysis process consisted of the following 5 iterative steps.

### Step 1. Familiarizing With the Data

The research team reviewed the verbatim transcribed data to ensure accuracy and a comprehensive understanding of the content. Repeated readings of the transcripts were conducted to immerse in the data and identify preliminary patterns relevant to stakeholders’ experiences and preferences for AURA.

### Step 2. Generating Initial Codes

Several researchers, including JL and DG, independently performed open coding to identify meaningful data segments related to stakeholder needs and preferences for AURA, which may shape AURA’s essential features and functionalities. Coding discrepancies were resolved through discussions among researchers (JL, DG, LS, and XW) to maintain consistency.

### Step 3. Identifying Recurring Themes

The UTAUT2 framework was used to guide thematic analysis. The UTAUT2 framework is an extension of the original UTAUT model, which was developed to examine technology acceptance in consumer contexts [31]. In UTAUT, there are 4 foundational constructs: performance expectancy, effort expectancy, social influence, and facilitating conditions, which collectively explain the drivers of technology adoption [29]. UTAUT2 builds on UTAUT and introduces 3 additional constructs (hedonic motivation, price value, and habit) to capture the nuanced factors influencing behavioral intention and technology use in voluntary, consumer-driven settings. For this study, we combined “hedonic motivation” and “habit” due to their conceptual overlap in this early-stage design context. Specifically, stakeholders’ expressions of emotional engagement and perceived enjoyment (eg, social interaction and mental stimulation) were closely tied to anticipated routine use. Since stakeholders interacted with a conceptual video rather than a functioning prototype, their responses reflected hypothetical experiences, and the distinction between intrinsic enjoyment and habit was not clearly articulated. We excluded “price value” because AURA has not yet been commercialized, and stakeholders were not evaluating its affordability.

Therefore, we focused on 5 constructs: performance expectancy, effort expectancy, social influence, facilitating conditions, and hedonic motivation and habit, to explore anticipated benefits, ease of use, external support, and emotional engagement (Table 1). This adapted framework was particularly relevant to examine stakeholder feedback on a home-based IoT system intended to support chronic illness management. This step was conducted primarily by researchers JL, DG, and others.

**Table 1:** Overview of UTAUT2^a^ constructs used for thematic analysis.

Constructs	Definition
Performance expectancy	Stakeholders’ perceptions of the benefits AURA^b^ could provide in supporting health outcomes and caregiving tasks
Effort expectancy	The system’s ease of use and perceived simplicity, particularly for patients with physical or cognitive challenges
Social influence	The impact of caregivers, clinicians, and social networks on the potential adoption of AURA
Facilitating conditions	The resources and infrastructure required to support the adoption and integration of AURA into daily care routines
Hedonic motivation and habit	The nuanced user preferences and potential adoption barriers

^a^UTAUT2: Unified Theory of Acceptance and Use of Technology 2.

^b^AURA: audio and radio connected.

### 4. Reviewing and Refining Themes

Identified themes were refined by researchers (JL, DG, and XW) to ensure consistency and alignment with stakeholder feedback. This iterative process involved reviewing themes for overlap, redundancy, and relevance to the study’s aim of informing the development of AURA. Any inconsistencies were resolved collaboratively among the research team members.

### 5. Defining and Naming Themes

Each theme was clearly defined in relation to the research aims and the UTAUT2 constructs by researchers (JL and DG), along with ongoing group discussion. Detailed descriptions and illustrative quotes were used to support and contextualize each theme, ensuring their interpretive robustness and relevance to AURA development.

### Ethical Considerations

The study was approved by the Institutional Review Board at the University of Texas Health Science Center at San Antonio (20210261HU and 20220750HU). All stakeholders signed consent forms before data collection. Every participating patient with cancer who had an ostomy received US $20 in compensation; every patient with ADRD and their caregiver received US $50.

## Results

### Patient Characteristics

The study included 11 patients, 6 of whom had cancer. The mean age was 78.7 (SD 6.6) years. The 6 patients with cancer who had an ostomy included 3 females and 3 males. Most patients (n=4) identified themselves as non-Hispanic White, while the remaining patients identified themselves as Hispanic. Marital status varied with 3 married, 1 widowed, and 2 divorced. Five patients had at least a bachelor’s degree, and 1 reported having completed high school. The types of ostomies among patients with cancer included urostomies (n=5) and colostomies (n=1).

Among the 5 patients with ADRD, 3 were female; all were non-Hispanic White, except for 1 male Hispanic patient; and 4 were married. Regarding education levels, all patients held at least a bachelor’s degree, and 3 had postgraduate degrees. Primary caregivers included spouses (n=4) and adult children (n=1). Detailed demographic information for patients is provided in Table 2.

**Table 2:** Patient characteristics.

Characteristic			Patients with cancer who had ostomy (n=6)		Patients with ADRD^a^ (n=5)
Age (years), mean (SD)			75.7 (4.7)		82.4 (7.1)
**Sex, n (%)**					
	Female	3 (50)		3 (60)	
	Male	3 (50)		2 (40)	
**Race, n (%)**					
	White	6 (100)		5 (100)	
**Ethnicity, n (%)**					
	Hispanic	2 (33)		1 (20)	
	Non-Hispanic	4 (67)		4 (80)	
**Marital status, n (%)**					
	Married	3 (50)		4 (80)	
	Widowed	1 (17)		1 (20)	
	Divorced	2 (33)		0 (0)	
**Education level, n (%)**					
	High school	1 (17)		0 (0)	
	Bachelor’s degree	3 (50)		2 (40)	
	Postgraduate degree	2 (33)		3 (60)	
**Type of ostomy, n (%)**					
	Urostomy	5 (83)		—^b^	
	Colostomy	1 (17)		—	
**Primary caregiver, n (%)**					
	Spouse	—		4 (80)	
	Adult child	—		1 (20)	

^a^ADRD: Alzheimer disease and related dementias.

^b^Not applicable.

### Major Themes of Stakeholders’ Needs and Preferences

This study investigated the needs and preferences of stakeholders regarding the envisioned AURA envisioned system, which was intended to support patients with physical and cognitive health challenges. Guided by the UTAUT2 framework, thematic analysis of stakeholder interviews identified 5 main themes. Table 3 presents a summary of stakeholders’ needs and preferences, with more detailed results for each theme provided below.

**Table 3:** Summary of stakeholders’ needs and preferences.

Major themes	Stakeholders’ needs and preferences	
	Patients with cancer	ADRD^a^ stakeholders
Performance expectancy	Prioritize physical health management (eg, activity tracking and bag change reminders) Emphasize EHR^b^ integration	Need support for routines (eg, medications, meals, and hydration) Emphasize emotional and social engagement
Effort expectancy	Feel comfortable with moderate complexity if usability is maintained Prefer practical, streamlined interfaces without unnecessary features	Require a simple, intuitive design Prefer 1- or 2-step voice commands to avoid cognitive overload
Social influence	Clinician endorsement builds trust	Caregivers are primary decision-makers Want features that ease caregiving, especially when caregivers are not present
Facilitating conditions	Barriers include physical fatigue and device maintenance Facilitators include customization and health care synchronization	Barriers include low-tech literacy and privacy concerns Facilitators include perceived caregiving support
Hedonic motivation and habit	Interested in motivation for physical activity and habit formation	Emphasize social connection, emotional engagement, and cognitive stimulation through interactive features

^a^ADRD: Alzheimer disease and related dementias.

^b^EHR: electronic health record.

#### Theme 1: Performance Expectancy

Stakeholders across both groups highlighted the potential benefits of the AURA system, including improved daily life and reduced caregiving burdens.

##### Needs and Priorities of Patients With Cancer

###### Physical Health Management

Patients with cancer who had ostomies focused on physical health management as their primary need. For these patients, practical features, such as activity tracking and ostomy bag change reminders, were seen as transformative. As 1 patient explained, “Tracking my activity levels and reminding me to change my bag before it’s too late would be life-changing.” Another similarly noted that “reminders for bag changes or tracking how long I’ve been active – those would make things so much easier.” Beyond task management, patients also described the emotional reassurance provided by passive monitoring, with 1 individual stating, “It’s reassuring to know something is monitoring me, especially when I’m on my own. It gives peace of mind.”

###### Electronic Health Record Integration

The ability to integrate with health care systems further enhanced the system’s perceived value. Patients expressed a desire for seamless communication with clinicians, with one noting, “Sharing my health data with my doctor could prevent unnecessary visits and give me more confidence in managing my care.” These comments also underscored how AURA could address physical and emotional needs.

##### ADRD Stakeholders’ Needs

###### Support for Routines

For ADRD stakeholders, the focus was on the system’s ability to support cognitive tasks and routines (eg, taking medications, eating meals, and keeping patients hydrated), which are essential for maintaining daily functioning and reducing caregiver burden. A caregiver explained, “It’s all about routine. If the system can help maintain that, it would take a load off me.” This highlights the pressing need for tools that not only assist in task management but also provide consistency in care. The importance of reminders for medications and meals was frequently mentioned, with caregivers appreciating how such features could help reduce their own stress. One caregiver shared, “If the system can remind them about medications and meals without waiting for them to ask, it would be a huge help.”

###### Emotional and Social Engagement

Beyond routine management, stakeholders saw potential in the system to foster emotional and social engagement. A community health worker noted, “If AURA could prompt him to talk to a family member or remind him to join an activity, it would really reduce his isolation.” This reflects the dual benefit of supporting caregiving tasks while also addressing patients’ emotional well-being, which stakeholders linked to improved QoL, noting that “something that keeps them engaged socially and emotionally would make a big difference.” Some even described the system as having the potential to become meaningfully integrated into daily life, with 1 stakeholder reflecting that “just having a system to be part of their day-to-day routine, like a family member almost, could be transformative.”

#### Theme 2: Effort Expectancy

Ease of use emerged as a critical consideration for stakeholders, though expectations differed between the 2 groups.

##### Patients With Cancer

###### Feeling Comfortable With Moderate Complexity as Long as Usability Is Maintained

Patients with cancer demonstrated more comfort with moderate complexity if the system delivered meaningful benefits, compared to ADRD stakeholders. A patient explained, “I’m okay learning how to use something if it really helps me keep track of my care.”

###### Preferring Practical, Streamlined Interfaces Without Unnecessary Features

While this group was open to learning, they still valued streamlined interfaces without unnecessary features. One patient stated, “ I don’t need all the bells and whistles. Just make it practical,” while another similarly remarked, “I don’t need a bunch of extras. Just give me a simple system that does the basics well.” These inputs reflect patients’ preference for a system that is simple, functional, and free of unnecessary features.

##### ADRD Stakeholders

###### Requiring a Simple, Intuitive Design

For patients with ADRD and their caregivers, simplicity was paramount. Stakeholders highlighted the importance of clear and intuitive design, as exemplified by the neuropsychologist's remark, “If it’s too complicated, they won’t use it. It needs to be as simple as turning on a light switch.” A caregiver echoed, “I don’t want to spend hours teaching them how to use it. It should just work.” This reflects the necessity for a system that minimizes cognitive strain for users with impairments.

###### Preferring 1- or 2-Step Voice Commands to Avoid Cognitive Overload

Voice interfaces were frequently mentioned as a solution, with stakeholders emphasizing the need for 1- or 2-step commands. One geriatrician explained, “If you overload them with commands or steps, it just won’t work. They’ll shut down.” This further explains that AURA can better serve individuals with limited technological experience by reducing complex commands.

#### Theme 3: Social Influence

Social influence refers to the impact of opinions and behaviors of nonpatient stakeholders on the decision to adopt and use AURA. In this study, stakeholders indicated that caregivers and clinicians significantly influenced patients’ decisions regarding AURA system adoption, particularly in guiding or endorsing its use.

##### Patients With Cancer: Clinician Endorsement Builds Trust

For patients with cancer, clinician endorsement was a significant factor in building trust and encouraging adoption. One patient remarked, “If my doctor tells me it’s useful, I’m more likely to trust it.” Another echoed this sentiment, noting, “Knowing my healthcare team can access the data makes me feel more confident.” Patients indicated that integration with their health care team increased their confidence in the system’s relevance and reliability.

##### ADRD Stakeholders

###### Caregivers Are Primary Decision-Makers

As the primary decision-makers, caregivers’ evaluations focused on how the system could significantly assist patients while alleviating their caregiving burden. One caregiver stated, “If it helps me manage their medications and appointments, I’ll absolutely support using it.” Stakeholders described caregivers as both end users and primary influencers in decisions about system use.

###### Wanting Features That Ease Caregiving, Especially When Caregivers Are Not Present

Caregivers also emphasized the importance of a system that could support patients when they were not physically present. As 1 caregiver explained, “Caregivers need something that can fill in the gaps when we’re not around.” Another similarly highlighted the shared benefit, noting that “a system that reminds them and keeps track of things when I’m not there—it would make a huge difference for both of us.” Stakeholders identified a need for features that support patient monitoring and task reminders when the caregiver is absent.

#### Theme 4: Facilitating Conditions

Barriers and facilitators to adoption varied across the 2 groups.

##### Patients With Cancer

###### Barriers Included Physical Fatigue and Device Maintenance

Patients with cancer focused more on physical barriers, such as fatigue and device maintenance. One patient explained, “Some days, I don’t have the energy to keep up with everything. The system needs to be low maintenance.”

###### Facilitators Included Customization and Health Care Synchronization

Facilitators for this group included customization and integration with health care platforms. A patient noted, “If it syncs with my doctor’s system and reminds me to do things like hydrate or exercise, that would be a game-changer.”

##### ADRD Stakeholders

###### Barriers Included Low-Tech Literacy and Privacy Concerns

ADRD stakeholders identified technological literacy and privacy concerns as significant barriers. Caregivers and health care providers frequently noted that older adults often struggled with technology. One health care provider remarked, “They’re not tech-savvy, so anything overly complicated won’t work.” Another added, “Technology is hard for older adults. If something goes wrong with the internet, they might not know how to fix it.” These challenges highlight the need for robust support systems and fail-safe mechanisms to address user concerns.

Privacy was another prominent concern, with stakeholders emphasizing the importance of data security. One caregiver stated, “I worry about who else might have access to the data. We want to feel safe, not monitored.”

###### Facilitators Included Perceived Caregiving Support

Despite these challenges, many stakeholders recognized the system’s potential. As 1 caregiver put it, “If it can help me manage daily routines, that’s worth overcoming the tech challenges.”

#### Theme 5: Hedonic Motivation and Habit

##### Patients With Cancer: Interested in Motivation for Physical Activity and Habit Formation

While both groups valued practical applications of the system, patients with cancer expressed an interest in features that encouraged physical activity. One patient shared, “Having a system reminds me to move, even if just a little, keeps me motivated to stay active.” Another added, “Something that motivates me to get up and move, even a little bit, would be great.” These responses reflect the potential for the system to support healthier habits and improve overall QoL.

##### ADRD Stakeholders: Emphasizing Social Connection, Emotional Engagement, and Cognitive Simulation Through Interactive Features

Emotional and social benefits were emphasized primarily by ADRD stakeholders. For this group, social engagement features were seen as essential. One health care provider explained that “sometimes they just need a reminder to call a family member or take a break—it helps them feel less isolated.” A caregiver similarly noted that “engagement features that give them something to look forward to would be wonderful.”

Stakeholders also highlighted the value of mental stimulation. One caregiver noted, “Features that promote mental stimulation, like games or conversation prompts, could really improve their mood and quality of life.” Stakeholders expressed interest in features that support emotional well-being by providing cognitively engaging and uplifting experiences.

## Discussion

### Principal Findings

This study explored stakeholders’ needs and preferences, primarily older adult patients aged 70 years and older with either physical or cognitive impairments, to inform the development of a preliminary smart IoT system, AURA, a user-centered solution designed to support care at home. Guided by the UTAUT2 framework, the thematic analysis revealed distinct yet complementary needs and preferences across 2 target populations—patients with physical and cognitive challenges. Additionally, valuable insights from health care providers, community health workers, and computer scientists enriched the understanding of user expectations and system design considerations.

The findings highlight distinct differences in stakeholder priorities between the 2 populations. Patients with cancer who had ostomies focused on features that assisted with physical self-care tasks, such as activity monitoring, hydration reminders, and managing their ostomy bags. In contrast, ADRD stakeholders primarily emphasized support for memory, routine management, and emotional well-being, reflecting the cognitive challenges of this group. These differences underscore the need for a flexible, adaptable system design that accommodates both physical and cognitive impairment care needs, while offering valuable guidance for optimizing AURA’s design and functionality to ensure its acceptability and effectiveness.

Unlike many existing IoT studies focused solely on older adults or specific technologies (eg, wearable sensors [32] and fall detection [33]), our study explores a broader range of user needs across 2 populations and highlights their preferences that cut across physical and cognitive domains. While some studies focus on technical feasibility, few engage patients and caregivers at this formative stage to inform the development process [34]. This stakeholder-informed approach adds value to the design of inclusive, usable technologies.

### Performance Expectancy

Stakeholders across both groups recognized the potential of AURA to support ADLs and reduce caregiving burdens, but their needs and preferences differed significantly. Patients with cancer who had ostomies prioritized physical health management, particularly features such as activity tracking, fall detection, and reminders for ostomy bag maintenance. Integration with health care systems was also a key expectation, reflecting the importance of seamless communication with clinicians to improve care coordination. Interestingly, this group placed less emphasis on emotional support, diverging from the literature that underscores the psychosocial needs of cancer survivors [35]. These findings suggest that patients with cancer who have undergone surgery may prioritize practical, health-focused functionalities over emotional engagement tools.

Conversely, for ADRD stakeholders, the system’s ability to provide cognitive support through reminders for medications, meals, and activities was critical. These findings are consistent with the systematic review, which suggests that the ability of passive monitoring and assistive technologies to provide immediate and tangible benefits, such as enhanced safety and support for daily routines, facilitates acceptance and adherence [36]. Emotional well-being and social engagement also emerged as important findings, with stakeholders emphasizing the need for features that combat isolation and foster interaction.

### Effort Expectancy

Ease of use was universally important, yet stakeholders’ expectations differed. Patients with cancer demonstrated a higher tolerance for a complex system as long as it effectively met their health management needs. Although they also favored practicality and intuitive interfaces, this group prioritized a balance between essential functions and ease of use. In contrast, ADRD stakeholders emphasized the need for simplicity, with a preference for intuitive design and minimal steps to minimize cognitive strain. They also preferred voice commands and intuitive interfaces as key facilitators and worried that complex systems would likely result in disengagement. These findings not only align with studies showing that simplicity and ease of use are critical to technology adoption among older adults [37] but also highlight the need for a flexible system design that adapts to varying user capabilities and expectations.

### Social Influence

The stakeholders emphasized the crucial role of caregivers and clinicians in influencing the acceptance and adoption of the AURA system. For patients with cancer, clinician endorsement and integration with health care platforms emerged as critical facilitators in building trust and encouraging system adoption. Integrating AURA with health care systems was viewed as critical to enhancing its perceived value and credibility, underscoring the importance of clinician-patient collaboration in chronic disease management [38].

For ADRD stakeholders, caregivers often act as gatekeepers and decision-makers, underscoring the importance of designing a system that alleviates caregiving burdens and enhances patient care and well-being. They emphasized the importance of features that could provide continuity of care when they are unavailable. This aligns with existing research emphasizing the central role of caregivers in technology adoption for patients with dementia [39].

### Facilitating Conditions

Barriers to adopting the AURA system varied across the 2 groups. Patients with cancer highlighted barriers, including physical fatigue and the complexity of device maintenance. Customization options and integration with health care platforms were identified as key facilitators of system adoption, consistent with research emphasizing the value of interoperability in health care technologies [40]. ADRD stakeholders identified technological literacy and privacy concerns as significant challenges. Older adults often struggle to manage complex technology, and privacy concerns related to voice-assistive devices, such as Alexa, were particularly common [41].

Despite these barriers, stakeholders acknowledged the system’s potential to support caregiving when caregivers are unavailable and to improve routine care management. These findings highlight the importance of designing AURA to be minimally demanding, both physically and cognitively, by incorporating intuitive interfaces, a simple setup, and reliable passive-sensing features. Privacy safeguards must be clearly communicated and customizable to address user concerns. Facilitators such as integration with existing health care systems, perceived caregiving support, and the ability to tailor the system to individual routines suggest that AURA should be flexible, interoperable, and responsive to user needs. Addressing these factors early in the design process will be essential to enhancing adoption, especially among older adults and individuals with limited digital literacy.

### Hedonic Motivation and Habit

Hedonic motivation and habit, defined as the degree of pleasure or enjoyment derived from using technology, emerged as a meaningful factor in the adoption of AURA, particularly among stakeholders caring for patients with ADRD. Patients with cancer tended to focus less on hedonic motivation and habits. Still, they appreciated features that encouraged physical activity and health tracking, which demonstrates the system’s potential to support physical health and healthy behaviors. While both groups valued practical applications, ADRD stakeholders particularly preferred features that support emotional well-being and social engagement to reduce isolation, highlighting the system’s potential to enhance QoL beyond basic caregiving tasks. The findings reveal that emotionally engaging features, such as social prompts, games, or personalized voice interactions, can foster positive user experiences and sustained use. These elements may enhance mood, reduce isolation, and improve adherence to care routines. Designing AURA with enjoyable and engaging components is therefore instrumental in ensuring long-term user engagement, especially in populations at risk of cognitive or emotional decline.

### Research Implications

The findings underscore the need for AURA to be adaptable and customizable to meet the distinct needs of older adults who manage physical and cognitive health challenges in home settings. For patients with cancer, advanced health tracking, customization, and seamless integration of health care are critical. For patients with ADRD and caregivers, simplified interfaces, proactive reminders, and privacy safeguards are essential. By integrating these features, the system can provide personalized, context-aware support that enhances independence, safety, and QoL.

The divergent preferences between these 2 patient populations suggest a fundamental trade-off in IoT system design: how to balance system simplicity (critical for cognitively impaired users and their caregivers) with the richer functionality desired by users with physical limitations but preserved cognitive capacity. Designing AURA to meet both needs requires modular interfaces or adaptive personalization features that adjust complexity based on user type or profile.

The contrast in effort expectancy between cancer patients and ADRD caregivers aligns with the UTAUT2 model but also reveals its limitation in capturing how cognitive capacity moderates perceived usability. Our findings suggest that cognitive impairment, rather than age or experience alone, is a key moderator that should be considered in future adaptations of UTAUT2 for health care settings.

The difference in hedonic motivation suggests that emotional engagement may not be a universal adoption driver across all chronic illness groups. While social stimulation is crucial for ADRD users, patients with cancer viewed motivation more behaviorally (eg, reminders to move). AURA’s design should therefore allow customization of motivational features based on user context, rather than assuming a one-size-fits-all engagement strategy.

### Limitations and Future Directions

The following limitations warrant consideration when interpreting the findings: (1) this study included only older adult populations managing ostomies for cancer treatment or ADRD in home settings; they were selected to capture physical versus cognitive health challenges that pose common challenges in chronic illness management. However, we recognize that these groups do not represent the broader spectrum of chronic conditions. As such, future research is needed to validate and expand these findings in other chronic illness populations to inform the scalability and broader applicability of smart IoT-based home care systems such as AURA.

Next, while the study provided rich, in-depth insights from stakeholders representing 2 older adult populations with distinct care needs related to self-management in home settings, the sample size was relatively small and lacked demographic diversity. Most stakeholders identified as non-Hispanic White and had high levels of education. However, as this was a formative, exploratory phase focused on understanding stakeholder needs and preferences for using IoT and voice-assistive technologies, the primary aim was to obtain detailed, user-centered input rather than achieve population-level representativeness. To address this limitation, the subsequent phase of the AURA project expanded recruitment to include stakeholders from diverse racial, ethnic, and socioeconomic backgrounds, thereby ensuring broader applicability and inclusivity in future development and testing.

Furthermore, this study used a preconstructed demonstration video to introduce the initial AURA concept during the semistructured interviews, as a fully developed prototype was not yet available. While this approach allowed stakeholders to visualize the proposed functionalities, it may have introduced social desirability or confirmation bias, as stakeholders might have shaped their responses in line with perceived expectations. However, the video presented generalized daily scenarios rather than idealized outcomes. Interview questions were neutrally worded and designed to elicit both positive and critical feedback, and interviewers avoided leading prompts. Future studies, including the pilot in the next phase, will involve deploying the actual AURA prototype at home to gather real-world usability and acceptability data.

Finally, this formative study did not include real-world testing of the AURA system and thus does not capture how user perceptions may evolve over time with actual system use. While stakeholders expressed enthusiasm for features such as reminders and activity tracking, this study did not assess measurable outcomes such as improvements in ADLs or reductions in caregiver burden. Therefore, although stakeholders perceived these features as beneficial, their actual impact remains to be evaluated. The next phase of the AURA project focuses on refining the prototype and conducting field usability testing. The research team has completed a home-based deployment study (phase 2) evaluating the usability, acceptability, and preliminary performance of AURA in real-world environments. Data analysis and the manuscript are currently under development. Future development will also emphasize cultural inclusivity, adaptive personalization based on user needs, and further integration with health care systems to support broader implementation.

### Conclusion

This study examined stakeholders’ perspectives on the initial concept of the smart IoT system AURA, designed to support older adults with chronic illnesses, specifically, patients with cancer who had an ostomy and patients with ADRD. Perspectives were also gathered from health care providers, community health workers, and computer scientists. By applying the UTAUT2 framework, this study revealed key differences and commonalities in stakeholder needs and preferences for using smart IoT and voice-assistive technologies in home-based care.

While the findings provide valuable insight to guide the early design of user-centered technology, several limitations should be acknowledged. The study sample was relatively small and lacked demographic diversity, which may limit the generalizability of results. Additionally, stakeholder feedback was based on conceptual demonstrations rather than real-world use, and outcomes such as feasibility, acceptability, or health impact were not directly measured.

Despite these limitations, this formative research lays the groundwork for developing scalable IoT solutions that can support ADLs, facilitate self-management, promote healthy behaviors, and reduce caregiver burden. Future work should focus on refining and prototyping the AURA system and evaluating its usability, acceptability, and effectiveness in real-world home settings.

## Abbreviations

ADL: activity of daily living
ADRD: Alzheimer disease and related dementias
AURA: audio and radio connected
CSI: Channel State Information
IoT: Internet of Things
PRISMS: Patient Reported Outcomes-Informed Symptom Management System
QoL: quality of life
UTAUT2: Unified Theory of Acceptance and Use of Technology

## Data Availability

The datasets generated or analyzed during this study are available from the corresponding author on reasonable request.
